# Internal and external factors affecting the performance score of surgical trainees doing laparoscopic appendectomy: a prospective, observational cohort study in a structured training programme

**DOI:** 10.1007/s00464-024-11007-2

**Published:** 2024-07-08

**Authors:** Benedicte Skjold-Ødegaard, Hege Langli Ersdal, Jörg Assmus, Kjetil Søreide

**Affiliations:** 1 Department of Gastrointestinal Surgery, Stavanger University Hospital, Stavanger, Norway; 2 Faculty of Health Sciences, University of Stavanger, Stavanger, Norway; 3 Department of Surgery, Haugesund Hospital, Haugesund, Norway; 4 Department of Anaesthesiology, Stavanger University Hospital, Stavanger, Norway; 5 Centre for Clinical Research, Haukeland University Hospital, Bergen, Norway; 6 Department of Clinical Medicine, University of Bergen, Bergen, Norway

**Keywords:** Laparoscopic appendectomy, Difficulty score, Proficiency, Simulation, Trainee, Trainer

## Abstract

**Background:**

Laparoscopic appendectomy is a common procedure and introduced early in general surgical training. How internal (i.e. surgeon’s experience) or external (i.e. disease severity) may affect procedure performance is not well-studied. The aim of this study was to evaluate factors that may have an influence on the performance scores for surgical trainees.

**Methods:**

A prospective, observational cohort study of laparoscopic appendectomies performed by surgical trainees (experience < 4 years) operating under supervision. Trainers evaluated trainees’ overall performance on a 6-point scale for proficiency. Perioperative data were recorded, including appendicitis severity, operating time and the overall difficulty of the procedure as assessed by the trainer. A “Challenging” procedure was defined as a combination of either/or “perforation” and “difficult”. Trainees who had performed > 30 appendectomies were defined as “experienced”. The trainees were asked if they had used simulation or web-based tools the week prior to surgery.

**Results:**

142 procedure evaluation forms were included of which 19 (13%) were “perforated”, 14 (10%) “difficult” and 24 (17%) “Challenging”. Perforated appendicitis was strongly associated with procedure difficulty (OR 21.2, 95% CI 6.0–75.6). Experienced trainees performed “proficient” more often than non-experienced (OR 34.5, 95% CI 6.8–176.5). “Difficult” procedures were inversely associated with proficiency (OR 0.1, 95% CI 0.0–0.9). In “Challenging” procedures, identifying the appendix had lowest proficiency (OR 0.4, 95% CI 0.1–0.9). The procedures assessed as “difficult” had significantly longer operating time with a median (IQR) of 90 (75–100) min compared to 59 (25–120) min for the non-difficult (*p* < 0.001).

**Conclusion:**

Both internal and external factors contribute to the performance score. Perforated appendicitis, technical difficult procedures and trainee experience all play a role, but a “difficult” procedure had most overall impact on proficiency evaluation.

**Graphical Abstract:**

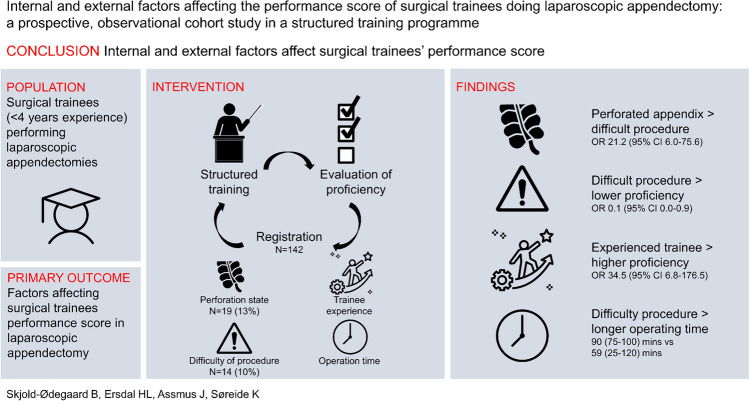

**Supplementary Information:**

The online version contains supplementary material available at 10.1007/s00464-024-11007-2.

Globally, there is a high life-time risk for developing appendicitis in the general population, making laparoscopic appendectomy one of the most frequently performed procedures in general surgery [1–3]. Appendectomy can be safely performed by trainees [4–6], and is integrated as part of most surgical curricula. However, knowledge about the factors that might impact trainee’s learning experience is scarce. The goal of surgical education is to bring the trainees to a level where performance is satisfactory [7, 8], however, there is a lack of a standardized definition on proficiency. Even commonly used terms such as “novice” and “expert” lack a clear definition [9] and this makes it difficult to compare different training methodologies and training programs.

Differentiating simple appendicitis from complicated appendicitis has become increasingly important regarding the use of conservative treatment of acute appendicitis [10]. Notably, the appendicitis disease state may also influence the surgical procedure, as a grossly inflamed or perforated appendicitis may be technical more difficult to operate on than a mildly inflamed appendix. Also, a retrocecal position of the appendix, or the patient’s habitus (i.e. obesity) may also contribute to the technical complexity of a procedure, of note, as appendectomies are an important procedure in the training of novice surgical residents, deeming the procedure as potentially difficult to perform can have implications for training, in particular when evaluating a non-standardized, non-simulated setting with real-life variation on a case to case setting. The severity of appendicitis varies from inflamed to gangrenous and perforated [11], and even a perforated appendicitis will inevitably vary from local inflammation via periappendiceal phlegmon or abscess up to generalized peritonitis with a grossly contaminated abdomen. Anatomical variations of the appendix could also influence the surgical procedure as some localizations of the appendix (for instance post-ileal) may make the identification more difficult. Patient factors could always have an impact on surgery, and higher age, obesity, higher ASA score (American Society of Anaesthesiologists physical Status Classification System) and diabetes have all been associated with higher case-complexity [6], and higher rate of conversion from laparoscopic to open appendectomy [12] and thus indicating more complicated procedures.

Some of these factors may be deemed as internal (i.e., trainee experience; subject to practice and modifiable) while others are external (i.e., grade of inflammation or anatomical conditions; less or non-modifiable factors.). Knowing how these factors may contribute to the performance evaluation during real-life surgery is not well-studied but may have an impact on trainee evaluation.

We hypothesized that both technical and disease-related factors may influence proficiency and thus need to be considered when evaluating training of surgical trainees. In particular for their proficiency evaluation in real-life, on patients with real diseases that may present on a spectrum from mildly to grossly inflamed, such as in the case of acute appendicitis. The aim of this study was to investigate internal as well as external factors, and how they might affect achievement of proficiency.

## Materials and methods

### Study ethics

The study was approved by the Regional Ethics Committee (REK 2018/811) and Data Protection Officer at Stavanger University Hospital. The current data were accrued through a prospective, observational cohort study and the STROBE recommendations [13] were followed, as applicable.

### Study population

The study cohort is based on a structured training program for laparoscopic appendectomy (LapApp) developed and implemented at Stavanger University Hospital in Norway. The training program is described in detail elsewhere [14–16]. The training programme consisted of an introductory course for the trainees and a train the trainer-course for the trainers, before implementing structured training and evaluation of each learning situation, that is, each real-time surgery where training took place. For the current cohort we included all consecutive procedures performed by surgical trainees (< 4 years of experience) for a 12-month period. Only procedures who were supervised and had evaluation scores completed were included. The trainers were either senior residents or consultant surgeons.

### Evaluation of procedures

For each appendectomy, the trainees were evaluated by the trainer on each of 8 predefined steps of the procedure, as well as on overall performance. The trainer evaluated the trainee on a 6-point scale from 1 (unable to perform) to 6 (expert performance) (supp 1). Proficiency was defined as the achievement of either 5 or 6 on overall performance on the scale. Scoring 1 or 2 on a procedural step means that the trainer has taken over that step. The operation time was registered for each procedure. Trainee and trainer gender were registered, as well as trainee experience and trainer experience. Trainee experience was defined as “low” or “high”, depending on whether the trainee had performed less or more than 30 appendectomies. Trainer experience was defined from the trainer being either senior resident or consultant surgeon. The trainees were asked to register whether they had used simulation tools and/or web-based learning tools the week prior to performing surgery. Operating time was registered for each procedure.

### Definitions of types of difficulty of procedure

We aimed to assess grade of difficulty in two ways. One, the technical difficulty as scored by the trainer and, second, by presence or not of a perforated appendix, assuming this would correlate with degree of inflammation and thus a more complicated procedure.

The difficulty of an operation was classified on a scale from 1 (very easy) to 6 (very difficult), and the trainer designated the difficulty score at the end of the procedure. For analysis, the “difficulty grade” was dichotomized into a “standard” (1-4) or “difficult” (5-6) category.

The appendix was described as either “non-perforated” or “perforated” based on gross evaluation by the trainee at the time of surgery.

Finally, the group of perforations and the procedures deemed “difficult” by the trainers were grouped together and named “Challenging” (as opposite to “Simple” which is procedures were neither the appendix is perforated nor the procedure was deemed “difficult”).

### Statistical analyses

Statistical analyses were done by SPSS v.26.0.0.1 (IBM SPSS; Armonk, NY, USA) and R 4.2.3 [17], while figures were computed in MathLab version 9 (Mathworks corp. Natick, MA). Descriptive methods were used to characterize the sample. Medians are reported with interquartile ranges (IQR), when appropriate. Fisher’s exact test were used to compute *p*-values in cross tabs due to the small numbers of data when analyzing subgroups. Unadjusted OR (odds ratio) was also reported.

All inference (confidence intervals, *p*-values, etc.) had to address the dependencies in the data caused by multiple measurements for both trainees and instructors. We did this using mixed effects models with random intercept for trainees and instructors. Otherwise, we abstained from inference to avoid biased results. The dependence of the proficiency (dichotomous) on the predictors was investigated using the logistic mixed effects model. The model was built in three steps. First, we estimate the univariate model for each predictor, second, we estimated the full model containing all predictors. Predictors with a *p*-value under 0.1 in at least 1 of the 2 first models were in the third step selected for the final model.

Using the same three steps we built the linear mixed model for the duration of surgery depending on proficiency, experience of the trainee, perforation and difficulty. All tests were 2-tailed and *p*-values < 0.050 were considered statistically significant.

## Results

**Fig. 1:**
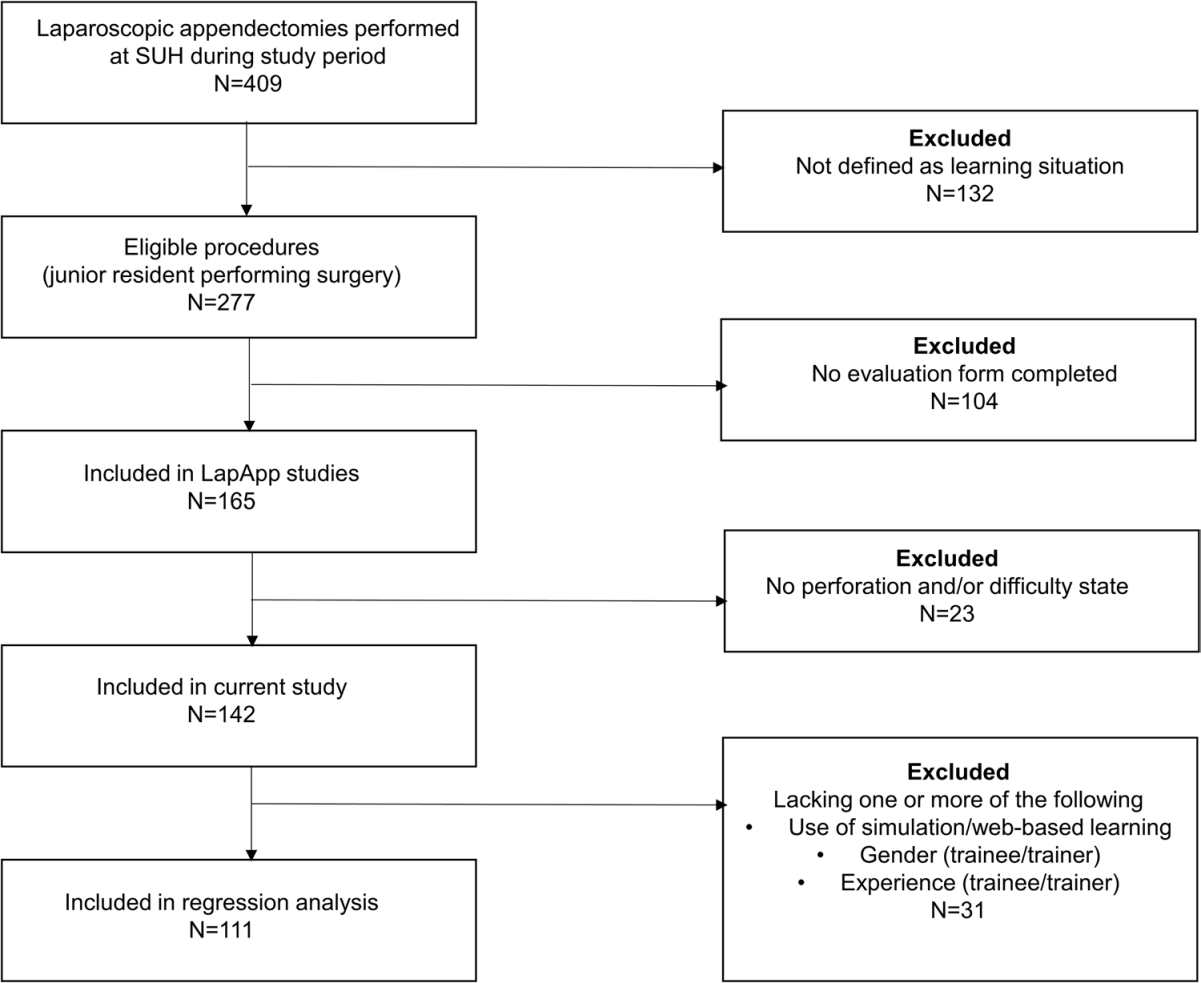
Study inclusion. Flowchart describing the study inclusion

During the study period, 409 appendectomies were performed and, 142 evaluation forms were completed and ready for data analysis (Fig. 1).

In 19 of 142 (13%) patients, the appendix was perforated. The assessment of difficulty for the procedures with either a non-perforated or the perforated appendicitis, is described in Table 1. A perforated appendix was associated with higher risk of having a difficult procedure.

**Table 1:** The difficulty score for the non-perforated and the perforated cases, and an overview over instructor experience

	Standard		Difficult	
	Total standard cases	% of cases where instructor is a senior trainee	Total difficult cases	% of cases where instructor is a senior trainee
Not perforated	118 (83%)	92 (78%)	5 (4%)	4 (80%)
Perforated	10 (7%)	9 (90%)	9 (6%)	9 (100%)
*n* (%) of procedures with instructor being a senior trainee	101 (79%)		13 (93%)	

To study the outcome of trainee achievement of proficiency, a regression analysis was performed. Potential influencing factors included in the analysis were; perforation of appendix (yes vs no), difficulty (standard vs difficult), use of simulation and web tools the week prior to performing surgery (yes vs no), gender of both trainee and trainer (female vs male) as well as experience of trainee (< 30 vs ≥ 30 procedures) and trainer (senior resident vs consultant surgeon) (Table 2). In the multivariable analysis (Table 2), procedure difficulty was inversely associated with the achievement of proficiency. The trainee’s prior experience was correlated with achievement of proficiency as the experienced trainees were more likely to be proficient.

**Table 2:** Logistic mixed effect model for achieving proficiency

	Univariate models			Full model		Final model	
	*n*	OR (95% CI)	*p*-value	*n* = 111		*n* = 145	
				OR (95%CI)	*p*-value	OR (95% CI)	*p*-value
Perforation (yes)	162	0.57 (0.14, 2.26)	0.426	2.6 (0.16, 42.42)	0.503	–	–
Difficulty (high)	145	0.1 (0.01, 0.87)	0.036	0.09 (0, 2.93)	0.177	0.14 (0.02, 0.89)	0.037
Use of simulations	126	0.75 (0.12, 4.69)	0.758	0.15 (0.01, 3.73)	0.250	–	–
Use of web-based learning	128	1.14 (0.19, 6.88)	0.887	6.25 (0.39, 99.71)	0.194	–	–
Gender trainee (male)	165	0.95 (0.08, 10.73)	0.966	1.79 (0.05, 66.49)	0.753	–	–
Gender instructor (male)	165	0.27 (0.04, 1.64)	0.154	0.03 (0, 1.62)	0.083	0.35 (0.06, 2.13)	0.255
Experienced trainee	165	34.03 (5.7, 203.11)	< 0.001	151.57 (3.81, 6032.49)	0.008	34.51 (6.75, 176.51)	< 0.001
Experience instructor (consultant)	165	1.31 (0.17, 9.95)	0.792	0.4 (0.01, 18.28)	0.640	–	–

**Fig. 2:**
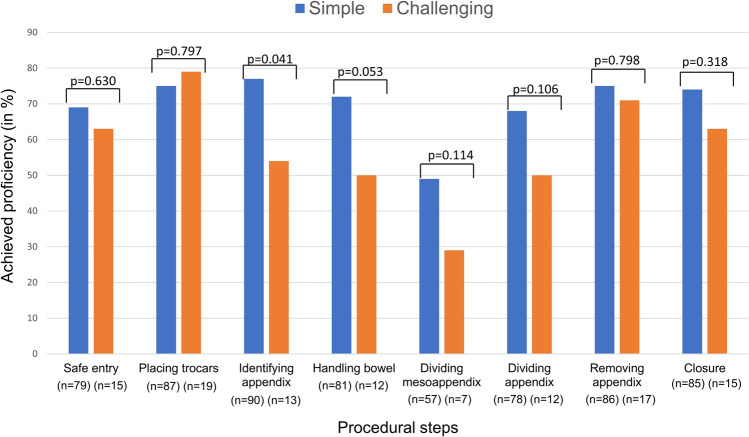
Proficiency on procedural steps. Achieved proficiency in (%) on each procedural step for simple and challenging procedures where challenging is defined as either the appendix being perforated and/or the procedure being deemed difficult by the trainer

The achieved proficiency on each procedural step was analysed (Fig. 2). The standard (“Simple”) procedures were compared to procedures defined as “Challenging”, either because the appendix was perforated and/or because the procedure was deemed difficult by the trainer. For the challenging cases, identifying the appendix achieved significantly lower proficiency.

**Fig. 3:**
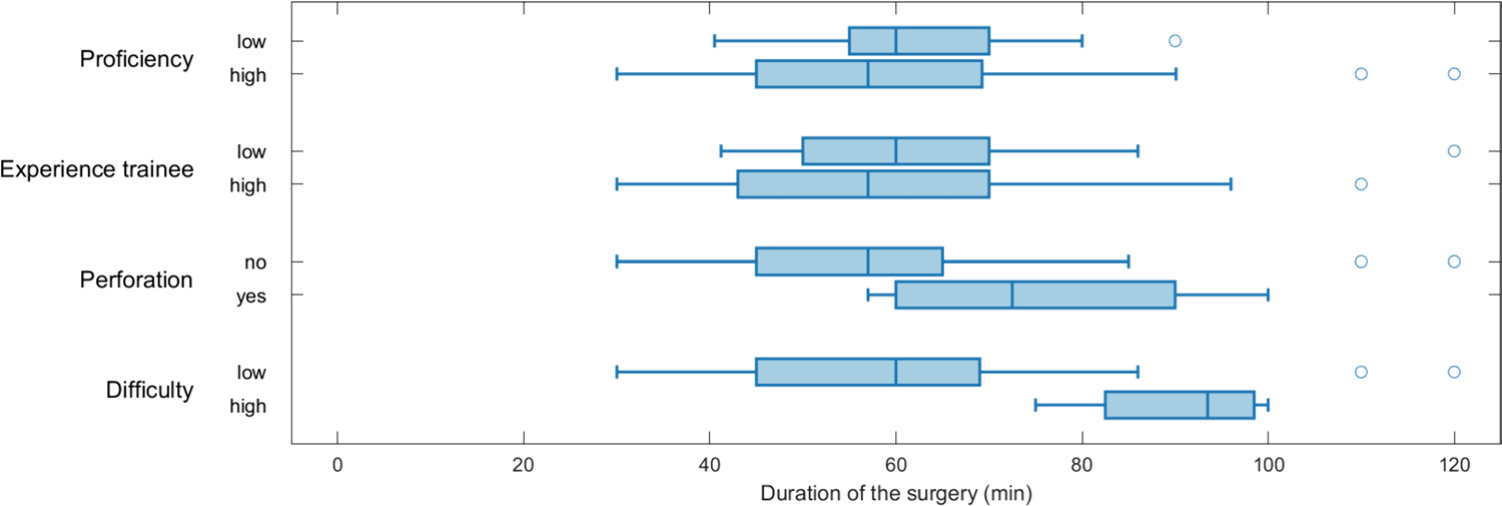
Operation time. Boxplot describing the operation time depending on four different variables

When analysing operating time, the procedures where the trainer had taken over one or more procedural steps were excluded. This led to an exclusion of 47 of 142 (33%) evaluation forms and 95 of 142 (67%) evaluation forms were included. The median (IQR) time of the procedure was analysed for different subgroups; trainee proficiency (proficient vs non-proficient), trainee experience (experienced vs not experienced), perforation state (perforated vs not perforated) and difficulty state (difficult vs standard). Boxplots for duration of operating time is illustrated in (Fig. 3). Only the definition of a “difficult” procedure led to a significant increase in the operating time (Table 3) with operating time 90 (75–100) min for the “difficult” procedures vs 59 (25–120) min for the non-difficult. Further analysis was performed to see if the experienced trainees took on more difficult cases. The appendectomy was deemed “difficult” in 9% of the cases when an experienced trainee performed surgery compared with 10% among the inexperienced trainees.

**Table 3:** Linear mixed model for the duration of surgery

	Univariate models			Full model	
	*n*	*B* (95% CI)	*p*-value	*n* = 82	
				*B* (95% CI)	*p*-value
Proficiency	95	− 6.5 (− 15.9, 2.9)	0.178	− 7.2 (− 17.3, 2.9)	0.164
Experience trainee	95	− 3 (− 12.3, 6.3)	0.538	− 2 (− 11.9, 7.9)	0.694
Perforation	93	19.6 (7.7, 31.4)	0.002	6.1 (− 8.2, 20.3)	0.407
Difficulty	84	37.6 (20.3, 54.9)	< 0.001	32.2 (10.1, 54.3)	0.006

## Discussion

This current study of surgical trainee’s performance found that training level and disease state influenced performance scores in real-life laparoscopic appendectomies. In assessing surgical competency, ideally one would include as many aspects as possible of the procedure [18]. Hence, both internal and external factors should be considered when evaluating surgical trainees performing a surgical procedure such as a laparoscopic appendectomy.

In this study we found an association between perforation state and the procedure being designated difficult by the trainer, yet only 9 (47%) of 19 perforated appendices were designated difficult. This suggests that disease severity influences on difficulty but that other factors than perforation may contribute to technical challenges during a procedure. Disease severity tends to be related to perforation state, i.e., such as proposed by the American Association for the Surgery of Trauma (AAST) grading system for acute appendicitis severity [19]. This is also consistent with the findings of a prospective observational trial [20] which demonstrated that the need for preoperative CT scan and a higher Appendicitis Inflammatory Response (AIR) score [21] resulted in a higher risk for a difficult appendectomy, defined as the need for help from an expert surgeon. Both the need for a preoperative CT and a higher Appendicitis Inflammatory Response (AIR) score will indicate a more advanced disease state.

The regression analysis demonstrated a very strong association between trainee experience and the achievement of proficiency. This association is in agreement with other studies that suggest a learning curve of 20–30 cases [22, 23] and thus trainee’s that have performed more than 30 cases would be considered proficient. However, studies indicate that improvement of surgical outcomes continue beyond the first 30 procedures [23].

Trainee’s performing a difficult procedure, had a significantly lower proficiency rate, compared to those who performed procedures not designated as difficult. From other studies, it is known that several factors can increase the risk of conversion from laparoscopic to open surgery and hence indicate a difficult procedure. These factors include severe inflammatory adhesions, high grades of appendiceal inflammation and perforation [24]. Patient anatomy, adhesions after prior surgery, obese patients and other patient factors can also increase the difficulty, and in a prospective observational trial [20], patient obesity (defined as Body Mass Index (BMI) > 30 kg/m^2^) resulted in a significant higher risk for a difficult procedure. Surgeon factors such as laparoscopic inexperience [24] also increases the risk for conversion from laparoscopic to open surgery. It is thus not surprising that inexperienced trainees will have a lower proficiency rate in difficult procedures—independent of the reason for the procedure being deemed difficult.

In a previous study [25] it was demonstrated that the chance of obtaining “proficiency” in a laparoscopic appendectomy was related to whether or not the trainee was able to complete each of the procedural steps. This current study further elaborates on how achieving proficiency also will depend on how challenging the procedure is—and this can depend on internal as well as external factors. Many of the contributing factors that play a role on performance cannot be controlled for but should be taken into consideration when deciding whether a patient planned for a procedure is suitable for a trainee to perform on. Particularly challenging steps of a procedure can be used as basis for developing specific training opportunities, i.e. by the use of simulation-based training. This will in turn enable deliberate practice—that is, training with focus on improving a particular task [26]—a prerequisite for competency-based surgical education [27].

Comparing the operating time in this study, we found no significant difference between the inexperienced and the experienced trainees. This stands in contrast with other studies [5, 28, 29], and a retrospective review [22] even demonstrated an almost linear reduction in operation time with increasing experience, which will indicate that the operative learning curve continues the first years after achievement of proficiency before reaching a plateau [6]. Operating time could also have implications for patient care as prolonged operating time is associated with an increased risk of postoperative complications [30]. However, in our current study cases that presented in an advanced disease state were not include as “training cases” in this real-time setting [31], and these cases have been demonstrated to increase operating time to such extend that one study even found that operating time was higher for trained surgeons than for trainees [6].

Previously we found an association between various procedure steps for laparoscopic appendectomy and the number of procedures required to achieve proficiency for any step [15]. The step involving division of the mesoappendix had the highest number of procedures required to achieve proficiency, and we suspect this may be related to varying degrees of inflammation possibly affecting the technical performance of the procedure. In this study, identifying the appendix had the lowest achievement of proficiency in the challenging cases.

The patients whose procedures were designated as “difficult” had significantly longer operating time than the “non-difficult” procedures (median 90 min vs 59 min; *p* < 0,001).

Some limitations to the study need to be mentioned. Due to the design of the study and the regulation of the regional ethics committee, no patient—nor procedure-specific information was recorded during the study. Hence, no recording of patient age, gender, body mass index, prior medical or surgical history was available. The anatomical description of the appendix was not reported, i.e. whether freely located in the pelvis or, alternatively, found as a retrocoecally placed organ. Variations in visceral fat, prior surgery or anatomical location could clearly have impacted on the procedure. Further, the procedure being designated as “difficult” was an individual judgement by the individual trainers. The trainers were not asked to register why the procedure was considered difficult (such as adhesions from previous surgery; visceral obesity; retrocoecally located appendix, or any other reason), and this combined with the individual judgement could represent a potential bias. For future studies, we believe these datapoints should be registered to better understand what makes a procedure being designated as “difficult”. Also, the trainers were either senior residents (more than 4 years of surgical training) or consultant surgeons with varying experience, and the trainer experience with laparoscopic surgery could have an influence on the individual judgement. An experienced trainee may also make the surgery appear less challenging due to his or her prior experience. Hence, we propose to make the “difficult” judgement more objective in the future with a formal assessment of what factors contribute to a difficult procedure.

Furthermore, the appendicitis severity was dichotomized into perforated and non-perforated, which may not reflect a more nuanced grade of disease impact on procedure performance. A more formal evaluation could have been entertained, with designation into more specific categories, such as the AAST severity score grading [11] to assess the local appendicitis situation. Others have evaluated the AAST severity score and validated its role in assessing severity for appendicitis [19, 32]. As disease severity may impact on procedure performance, it is important to consider this information as it may impact on the difficulty grade and the ability to master a procedure. Both patient-specific factors (age, gender, and body mass index, anatomical variation and prior surgery) and disease-specific factors (i.e., Appendicitis Inflammatory Response (AIR) score and AAST grading) should ideally have been registered in this study to better inform on the details of internal and external factors. While this was beyond the scope of the current project, future projects should address these items to help better understand the impact on training and performance.

The concept of “complicated appendicitis” has been thoroughly studied throughout the last decades. However, for both surgical trainees and trainers, the “complicated appendectomy” will be even more important to consider as external and internal factors will be an important confounder when evaluating trainee performance in a real-life setting. Even though it is not possible to control for disease severity, registering factors that constitutes a complicated appendectomy can give a better evaluation of proficiency among surgical trainees.

### Supplementary Information

Below is the link to the electronic supplementary material.

Supplementary file1 (DOCX 26 kb)
