# Supplementary material for: Internal and external factors affecting the performance score of surgical trainees doing laparoscopic appendectomy: a prospective, observational cohort study in a structured training programme
Source: Surg Endosc. 2024 Jul 8;38(9):4939–46. doi: 10.1007/s00464-024-11007-2 (PMC11362477; doi:10.1007/s00464-024-11007-2)
Supplement: Supplementary file 1 — Supplementary file1 (DOCX 26 kb) [file 464_2024_11007_MOESM1_ESM.docx]

Supplementary 1 Scoring system used by trainers and proficiency status

| 1 | Unable to perform | Non-proficient |
| --- | --- | --- |
| 2 | Partly performed | Non-proficient |
| 3 | Performed with substantial verbal support | Non-proficient |
| 4 | Performed with minor verbal support | Non-proficient |
| 5 | Performed independently | Proficient |
| 6 | Expert performance | Proficient |
